
JOURNAL INFORMATION
==============================
NLM Title Abbreviation: ScientificWorldJournal
Journal ID: ScientificWorldJournal
NLM Journal ID: 101131163
Journal ID: TSWJ

The Scientific World Journal

ISSN: 2356-6140
EISSN: 1537-744X
Publisher: Wiley

ARTICLE INFORMATION
==============================
PMCID: PMC5720074
PMID: 21380487
DOI: 10.1100/tsw.2011.53
Article ID: 350924
Article version: 1
Subjects: Editorial

Biometrics Applications: Technology, Ethics, and Health Hazards

Tzaphlidou Margaret 1 *
Pavlidou Fotini-Niovi 2
1 Department of Medical Physics, Medical School, Ioannina University, Ioannina, Greece
2 Department of Electrical and Computer Engineering, Aristotle University of Thessaloniki, Thessaloniki, Greece
*Margaret Tzaphlidou: mtzaphli@uoi.grAcademic Editor: Claire Redhead

Publication date: 2011
Electronic publication date: 2011 Mar 1
Volume: 11
First page: 529
Last page: 531
Received 2010 Dec 21; Revised 2011 Jan 19; Accepted 2011 Jan 20
Copyright: Copyright © 2011 Margaret Tzaphlidou and Fotini-Niovi Pavlidou.
Copyright year: 2011
License: This is an open access article distributed under the Creative Commons Attribution License, which permits unrestricted use, distribution, and reproduction in any medium, provided the original work is properly cited.
License URL: https://creativecommons.org/licenses/by/3.0/

==============================
Editorial
Special Issue: Biometrics Applications: Technology, Ethics, and Health Hazards
*TheScientificWorldJOURNAL* (2011) 11, 529–531
ISSN 1537-744X; DOI 10.1100/tsw.2011.53

TheScientificWorldJOURNAL
www.thescientificworld.com

### Biometrics Applications: Technology, Ethics, and Health Hazards

### Special Issue

Margaret Tzaphlidou[1,*] and Fotini-Niovi Pavlidou[2]

*[1]Department of Medical Physics, Medical School, Ioannina University, Ioannina Greece, [2]Department of Electrical and Computer Engineering, Aristotle University of Thessaloniki, Greece*

**E-mail:** mtzaphli@uoi.gr, niovi@auth.gr

*Received December 21, 2010; Revised January 19, 2011; Accepted January 20, 2011; Published March 1, 2011*

---

Biometrics is the science and technology of measuring and analyzing biological data for authentication purposes. Among the features measured are DNA, face, fingerprints, eye retinas and irises, hand geometry, handwriting, veins, and voice[1,2].

Biometrics has fast emerged as a promising technology for authentication and has already found a place in most hi-tech security areas. Biometric technologies are becoming the foundation of an extensive array of highly secure identification and personal verification solutions. As the level of security breaches and transaction fraud increases, the need for highly secure identification and personal verification technologies is becoming apparent[3]. Biometric-based solutions are able to provide for confidential financial transactions and personal data privacy.

However, a variety of ethical concerns with biometric identification methods have been registered by users and many questions have also arisen about how these data will be stored and used[4,5].

It is common knowledge that all biometric systems must potentially fulfill a number of certain properties. Among them, of great importance are the requirements for safety and privacy protection that constitute a source of fear and unwillingness towards biometric technologies. "User acceptance" of a biometric transaction is directly correlated with the medical implications resulting from such a process, excluding parameters such as psychology and ergonomics[6,7].

This special issue of *TheScientificWorldJOURNAL* intends to present today's international work on the subject, to give particular answers and solutions, and to provide the bases for further investigation.

The paper by Tsoukalas and Siozos[8] addresses the biggest risks facing today's Information Society due to the concept of privacy and data protection. According to the authors, in this context, the EU needs to work towards a "Privacy by Education" approach for the empowerment of "privacy-literate" European digital citizens. Leavitt[9] discusses whether or not biometrics and data sharing are consistent with democracy. In addition, he does not reject the idea of including religious, philosophical, and ideological data in a biometric profile. Nevertheless, the contribution of Nouskalis[10] states that the data processing based on biometrics is covered both by Directive 95/46 EC and Article 8 of the European Convention on Human Rights (ECHR).

The large-scale use of electronic biometric documents, like the recently introduced electronic passport (e-Passport), can cause privacy implications and threats to surface. Vakalis[11] presents the technical characteristics of the e-Passport, including the digital data structure, the communication, and reading mechanisms, indicating the possible points and methods of attack. Furthermore, de Hert and

*Corresponding author.
©2011 with author.
Published by TheScientificWorld; www.thescientificworld.com

Bellanova[12] question the modes of implementation of some of the most relevant technologies already operating at the borders: body scanners, biometrics, registered travellers' programs, and passenger name records. These authors propose to combine insights and solutions of both consumer and data protection law, in order to offer travellers a more "consumer friendly" approach to border check technologies. Towards a modular, robust, multimodal biometrics security authentication and monitoring system, Drosou et al.[13] describe a system that utilizes a biodynamic physiological profile, unique for each individual, and advancements in unobtrusive behavioral and other biometrics, such as face, gait recognition, and seat-based anthropometrics.

Up to now, two of the most promising biometric modalities are iris and retina recognition, which primarily use nonionizing radiation in the infrared region. Although only few infrared light emitting diodes are capable of causing direct eye damage, there is a growing concern about the possible use of such arrays that might pose a potential threat. Kourkoumelis and Tzaphlidou[14] explore the biological effects arising from exposing the eye to near infrared radiation, with reference to international legislation.

In conclusion, in the name of our safety during travelling, the perspective of the citizen should not be forgotten. Electronic information is challenging traditional views on property and privacy. Data security and protection of privacy should have high priorities in our societies. The involvement of possible biological effects arising from the use of biometrics could be a real scenario.

### ACKNOWLEDGMENTS

We sincerely thank all the authors for their contribution to this special issue. We also thank Dr. N. Kourkoumelis for his valuable help in the preparation of this issue.

### REFERENCES

1. Serrano, A., de Diego, I.M., Conde, C., and Cabello, E. (2010) Recent advances in face biometrics with Gabor wavelets: a review. *Pattern Recognition Lett.* **31**, 372–381.
2. Wang, L., Leedham, G., and Choa, D.S.Y. (2008) Minutiae feature analysis for infrared hand vein pattern biometrics. *Pattern Recognition* **41**, 920–929.
3. Du, Y.Z. and Chang, C.I. (2008) 3D combinational curves for accuracy and performance analysis of positive biometrics identification. *Optics Lasers Eng.* **46**, 477–490.
4. Mordini, E. and Massari, S. (2008) Body, biometrics and identity. *Bioethics* **22**, 488–498.
5. Wickins, J. (2007) The ethics of biometrics: the risk of social exclusion from the widespread use of electronic identification. *Sci. Eng. Ethics* **13,** 45–54.
6. Flores Zuniga, A.E., Win, K.T., and Susilo, W. (2010) Biometrics for electronic health records. *J. Med. Syst.* **34**, 975–983.
7. Kourkoumelis, N. and Tzaphlidou, M. (2010) Medical safety issues concerning the use of incoherent infrared light in biometrics. *Lect. Notes Comput. Sci.* **6005,** 121–126.
8. Tsoukalas, I.A. and Siozos, P.D. (2011) Privacy and anonymity in the information society – challenges for the European Union. *TheScientificWorldJOURNAL* **11,** 458–462.
9. Leavitt, F.J. (2011) Democracies restricting democratic rights: some classical sources and implications for ethics of biometrics. *TheScientificWorldJOURNAL* **11,** 463–473.
10. Nouskalis, G. (2011) Biometrics, e-identity, and the balance between security and privacy: case study of the Passenger Name Record (PNR) system. *TheScientificWorldJOURNAL* **11,** 474–477.
11. Vakalis, I. (2011) Privacy and biometric passports. *TheScientificWorldJOURNAL* **11,** 478–489.
12. De Hert, P. and Bellanova, R. (2011) Mobility should be fun. A consumer (law) perspective on border check technology. *TheScientificWorldJOURNAL* **11,** 490–502.
13. Drosou, A., Ioannidis, D., Moustakas, K., and Tzavaras, D. (2011) Unobtrusive behavioral and activity-related multimodal biometrics: the ACTIBIO authentication concept. *TheScientificWorldJOURNAL* **11,** 503–519.
14. Kourkoumelis, N. and Tzaphlidou, M. (2011) Eye safety related to near infrared radiation exposure to biometric devices. *TheScientificWorldJOURNAL* **11,** 520–528.

**This article should be cited as follows**:

Tzaphlidou, M. and Pavlidou, F.-N. (2011) Biometrics applications: technology, ethics, and health hazards. Special issue. *TheScientificWorldJOURNAL* **11,** 529–531. DOI 10.1100/tsw.2011.53.
